# The Relationship Between Human Cerebrospinal Fluid Proteins and the Risk of Delirium: A Study Based on Genetic Data

**DOI:** 10.1002/brb3.70836

**Published:** 2025-09-09

**Authors:** Zhihui Xu, Fei Ye, Zhantang Yuan, Chiyi Liu, Simin Zhu, Binfei Li, Qibiao Wu

**Affiliations:** ^1^ Faculty of Chinese Medicine Macau University of Science and Technology Macau China; ^2^ Department of Emergency Medicine Zhongshan Stomatological Hospital Zhongshan City Guangdong China; ^3^ Department of Anesthesiology Zhongshan People's Hospital Zhongshan City Guangdong China; ^4^ Chinese Medicine Guangdong Laboratory (Hengqin Laboratory) Guangdong‐Macao Ln‐Depth Cooperation Zone in Hengqin Hengqin China; ^5^ Zhuhai MUST Science and Technology Research Institute Zhuhai China

**Keywords:** cerebrospinal fluid proteins, cytokine signaling pathway, delirium, Mendelian randomization

## Abstract

**Background:**

Delirium is an acute cognitive disturbance that is linked to increased healthcare costs, extended hospitalization, and a greater incidence of adverse outcomes, including cognitive decline. Despite its clinical importance, existing strategies for predicting and managing delirium remain inadequate. This study, therefore, sought to investigate the potential relationship between cerebrospinal fluid proteins and delirium via Mendelian randomization (MR) and to identify potential therapeutic targets.

**Methods:**

Genetic data related to delirium were obtained from the 11th iteration of the FinnGen Biobank, which includes a total of 431,880 individuals of Finnish ancestry consisting of 3827 cases and 428,053 controls. Data on 910 cerebrospinal fluid proteins from 970 samples were collected via the ONTIME platform (https://ontime.wustl.edu/hg38/). MR analysis was used to evaluate genetic associations between cerebrospinal fluid proteins and delirium. Additionally, enrichment analysis was performed on cerebrospinal fluid proteins with genetic associations to identify potential cellular pathways and therapeutic targets.

**Results:**

We identified 46 cerebrospinal fluid proteins associated with the occurrence of delirium. Among these, insulin (odds ratio [OR]: 1.35, 95% confidence interval [CI]: 1.07–1.70, *p* = 0.01), interleukin‐7 (OR: 0.56, 95% CI: 0.37–0.85, *p* = 0.01), and B‐cell lymphoma/leukemia 2‐like protein 1 (OR: 0.63, 95% CI: 0.45–0.88, *p* = 0.01) were identified as key proteins. Horizontal pleiotropy had a minimal impact on establishing causal relationships, with *p* values of 0.08, 0.26, and 0.32, respectively. Additionally, no evidence of heterogeneity in genetic variation was found between these three cerebrospinal fluid proteins and delirium, with *p* values of 0.07, 0.45, and 0.96, respectively. Leave‐one‐out analysis further confirmed the stability and robustness of these associations. The enrichment analysis indicated that the cytokine‐mediated signaling pathway plays a significant role in the pathogenesis of delirium.

**Conclusion:**

Our study identified a genetic causal relationship between specific cerebrospinal fluid proteins and delirium, with insulin being a key factor. We also found that cytokine‐mediated signaling pathways may contribute to the pathophysiology of delirium. Future research should focus on the roles of peripheral and central glucose metabolism, as well as cellular immunity, in the pathological processes of delirium.

## Graphical abstract

This graphical abstract summarizes a Mendelian randomization study exploring the causal relationship between cerebrospinal fluid (CSF) plasma proteins and delirium.

**1**. **Data Sources**: The study uses 910 CSF plasma proteins as exposures, sourced from the ONTIME database, and examines their associations with delirium outcomes (non‐induced by alcohol or other psychoactive substances) using genetic data from FinnGen (3,827 cases and 428,053 controls).

2. **Statistical Analysis**: Various Mendelian randomization methods are applied, including weighted median, weighted mode, simple MR‐Egger, and inverse variance weighted methods. Sensitivity analyses include heterogeneity and pleiotropy assessments using the MR‐Egger intercept test and Q‐statistic test to ensure the robustness of results.

3. **Pathway Analysis**: Protein‐protein interaction networks and KEGG pathway enrichment analyses are conducted to identify inter‐protein relationships and key biological pathways involved.

This workflow highlights the integration of genetic data, advanced statistical analyses, and pathway enrichment to investigate the molecular mechanisms underlying delirium.

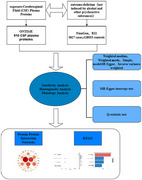

## 1 Introduction

Delirium is an acute neuropsychiatric syndrome characterized by alterations in consciousness, attention, cognition, and mood. According to the Diagnostic and Statistical Manual of Mental Disorders, Fifth Edition, Text Revision (DSM‐5‐TR), delirium can be classified into hyperactive, hypoactive, and mixed subtypes on the basis of activity level (First et al. 2023).

The incidence of delirium in elderly patients undergoing elective surgery during hospitalization varies between 22% and 50%. This condition is frequently associated with various factors, including medications, infections, and metabolic disturbances (Marcantonio 2017).

Delirium is associated with adverse outcomes such as prolonged hospitalization, increased risk of dementia, and increased mortality rates (Wu et al. 2019; Liang et al. 2021). Given the limited efficacy of current pharmacological treatments, which focus primarily on symptom management and address underlying conditions, there is an urgent need to identify risk factors and potential therapeutic targets.

Research has demonstrated that specific cerebrospinal fluid (CSF) proteins can serve as biomarkers for neurological damage and degenerative diseases. For example, the neurofilament light chain (NfL) in CSF is elevated in various central nervous system disorders, including Alzheimer's disease, Parkinson's disease, and amyotrophic lateral sclerosis, and is thought to be associated with axonal damage (Bridel et al. 2019). Additionally, the expression of FK506‐binding protein 4 (FKBP4) is significantly altered in the CSF and midbrain dopaminergic neurons (mDAs) of patients with the GBA1 variant Parkinson's disease (GBA1‐PD) (Kojima et al. 2024). Therefore, this study aimed to investigate the possible causal associations between CSF proteins and the occurrence of delirium and to explore the potential pathological mechanisms underlying this condition.

Advances in genomics and proteomics have significantly enhanced our understanding of the genetic factors and biomarkers associated with delirium. High‐throughput genomic technologies have facilitated the identification of genetic susceptibility loci linked to delirium risk, such as the ε4 allele of the APOE gene (Sepulveda et al. 2021), which some studies have associated with an increased risk of delirium, particularly in older populations. However, the relationship between APOE and delirium remains inconclusive, likely due to variations in study designs and population heterogeneity, which may be influenced by sample differences and environmental factors. Concurrently, proteomics advancements have enabled detailed analyses of biomarkers in CSF, including neurofilament light chain (NfL) (Bridel et al. 2019), which is elevated in several central nervous system disorders such as Alzheimer's disease and Parkinson's disease, where it is closely tied to axonal damage. Furthermore, alterations in FKBP4 in the CSF (Kojima et al. 2024), particularly in genetic contexts like GBA1 mutation carriers in Parkinson's disease, uncover mechanisms linked to neurodegenerative processes, offering new insights into the potential role of these protein changes in the pathophysiology of delirium. Therefore, this study aimed to investigate the genetic associations between CSF proteins and the occurrence of delirium and to explore the potential pathological mechanisms underlying this condition.

Mendelian randomization (MR) (Skrivankova et al. 2021) is a causal inference method that uses genetic variation to assess causal relationships. By capitalizing on the random allocation of genotypes at birth, MR effectively mitigates the influence of confounding variables and reverse causation, allowing for more accurate evaluations of causal links between environmental factors and disease outcomes. In this method, genetic variants act as instrumental variables (IVs) for exposure factors. In this study, we integrated delirium data from the Finnish Biobank with protein data from CSF samples collected via the ONTIME platform to perform an MR analysis and explore the potential impact of CSF proteins on delirium.

## 2 Materials and Methods

### 2.1 MR Analysis

**FIGURE 1:**
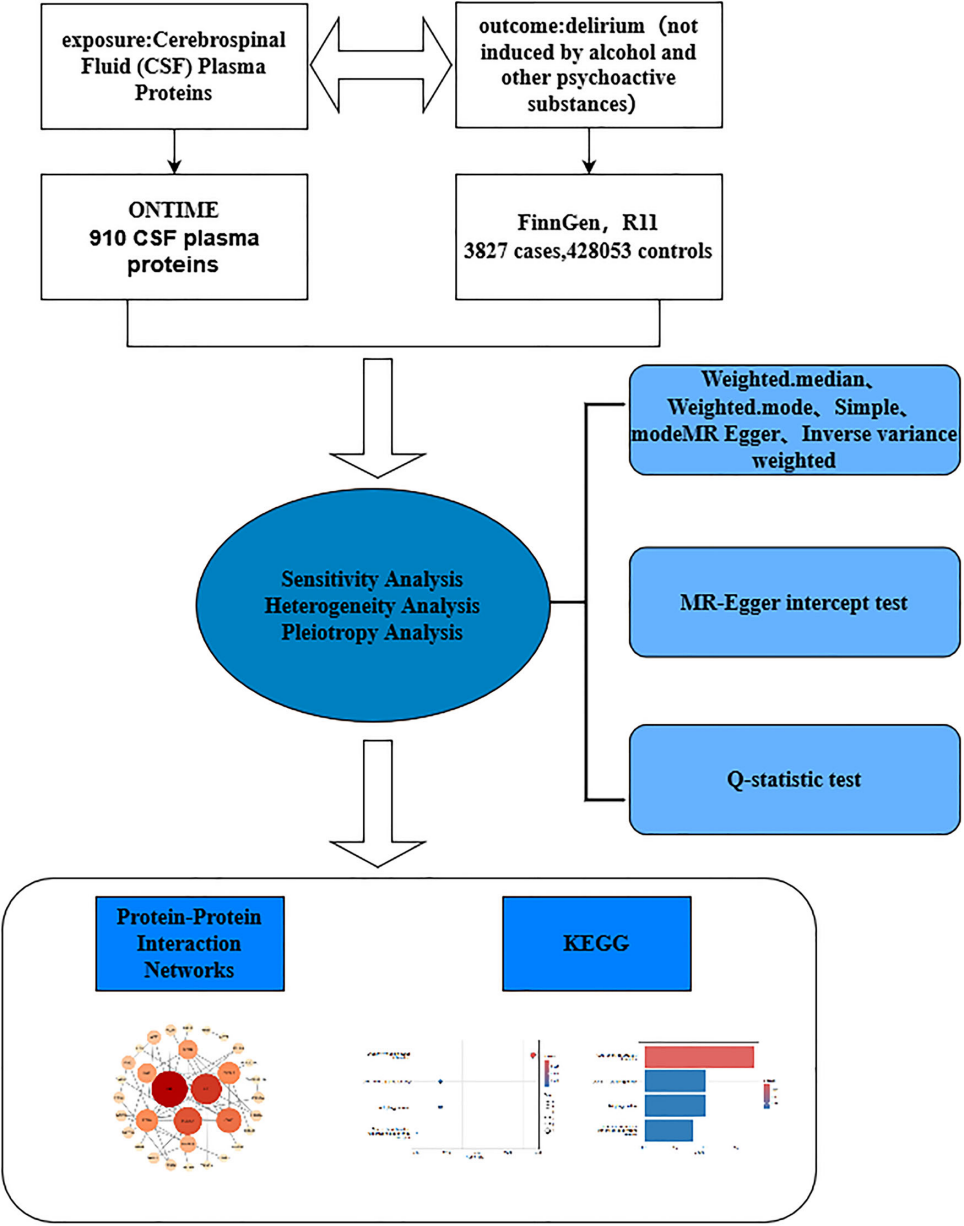
The study design.

This study employed five distinct statistical methods, including inverse variance weighting (IVW), MR‐Egger regression, the simple model, weighted median, and weighted mode, to investigate the genetic relationship between CSF proteins and delirium. We used Cochran's Q statistic to assess heterogeneity among the genetic instruments in the MR analysis. In the scatter plot, an MR‐Egger model intercept value close to zero indicates minimal horizontal pleiotropy; however, its statistical significance must be confirmed through appropriate tests. The slope's positive or negative direction reflects the causal effect's direction (positive or negative) but requires validation through statistical significance and biological relevance. Furthermore, in the funnel plot, a symmetric distribution of instrumental variables (IVs) suggests limited pleiotropy or bias, indirectly supporting the validity of the second assumption of MR. The pleiotropy test was performed via MR‐PRESSO and MR‐Egger intercept tests, whereas the leave‐one‐out method was utilized to assess the impact of individual SNPs on the results, further validating the robustness of the findings. The study design is illustrated in Figure 1.

Figure 1 shows a MR study exploring the link between 910 CSF plasma proteins and delirium, using genetic data from FinnGen and ONTIME. It employs various statitical analyses, builds protein interaction networks, and performs KEGG pathway analysis to understand biological processes.

### 2.2 Data Sources

Data on delirium were obtained from the FinnGen consortium (https://r11.finngen.fi/). The FinnGen database integrates samples from Finnish biobanks with phenotypic data from national health registries. For this study, we used the most recent version (FinnGen R11), which includes 3827 cases and 428,053 controls, with all cases identified using ICD‐10 code F05 and its subtypes, to assess GWAS summary statistics related to delirium.

The protein data for CSF were obtained from the study by Yang (Yang et al. 2021), published on the ONTIME platform (https://ontime.wustl.edu). This study utilized CSF samples and quantified protein abundance using an aptamer‐based high‐throughput platform. The expression levels of 1305 proteins were measured across 971 CSF samples. To ensure the reliability of the data, we implemented rigorous quality control measures on the downloaded protein expression data. These steps included: verifying the limit of detection (LOD) for each protein to ensure that all measurements exceeded the detection threshold; calculating the coefficient of variation (CV) for each protein and excluding those with a CV greater than 0.15; and applying the interquartile range (IQR) method to identify and exclude outliers. Following these procedures, high‐quality expression data for 910 proteins were retained.

### 2.3 Selection of Instrumental Variables

In this study, we utilized a rigorously reviewed instrumental variable approach to investigate the genetic relationship between exposures and outcomes. Given the limited number of single‐nucleotide polymorphisms (SNPs) meeting genome‐wide significance thresholds in current genome‐wide association study (GWAS) summary data, we adjusted the significance threshold to *p* < 5 × 10^−6^ (*F* > 10) to identify SNPs strongly associated with exposures. To mitigate linkage disequilibrium among instrumental variables, we performed clumping with specific parameters (*r^2^* < 0.1, clumping distance = 500 kb). Additionally, we excluded palindromic SNPs with intermediate allele frequencies to ensure that the SNPs’ effects on both exposures and outcomes were mediated by the same alleles. These measures were implemented to increase the accuracy and reliability of the study findings.

### 2.4 Statistical Analysis

All the statistical analyses were performed via R software (version 4.3.3) and the TwoSampleMR package (version 0.5.7), with a significance threshold set at *p* < 0.05. A protein‒protein interaction (PPI) network for the protein‒coding genes was constructed via the STRING database (https://cn.string‐db.org) and visualized with Cytoscape software (version 3.10.0) to explore protein‒level interactions. Kyoto Encyclopedia of Genes and Genomes (KEGG) enrichment analysis was carried out via the cluster Profiler package in R (version 4.10.1), with pathways considered significantly enriched at *p* < 0.05. This approach provided further insights into the biological functions and relationships of these proteins.

## 3 Results

**FIGURE 2:**
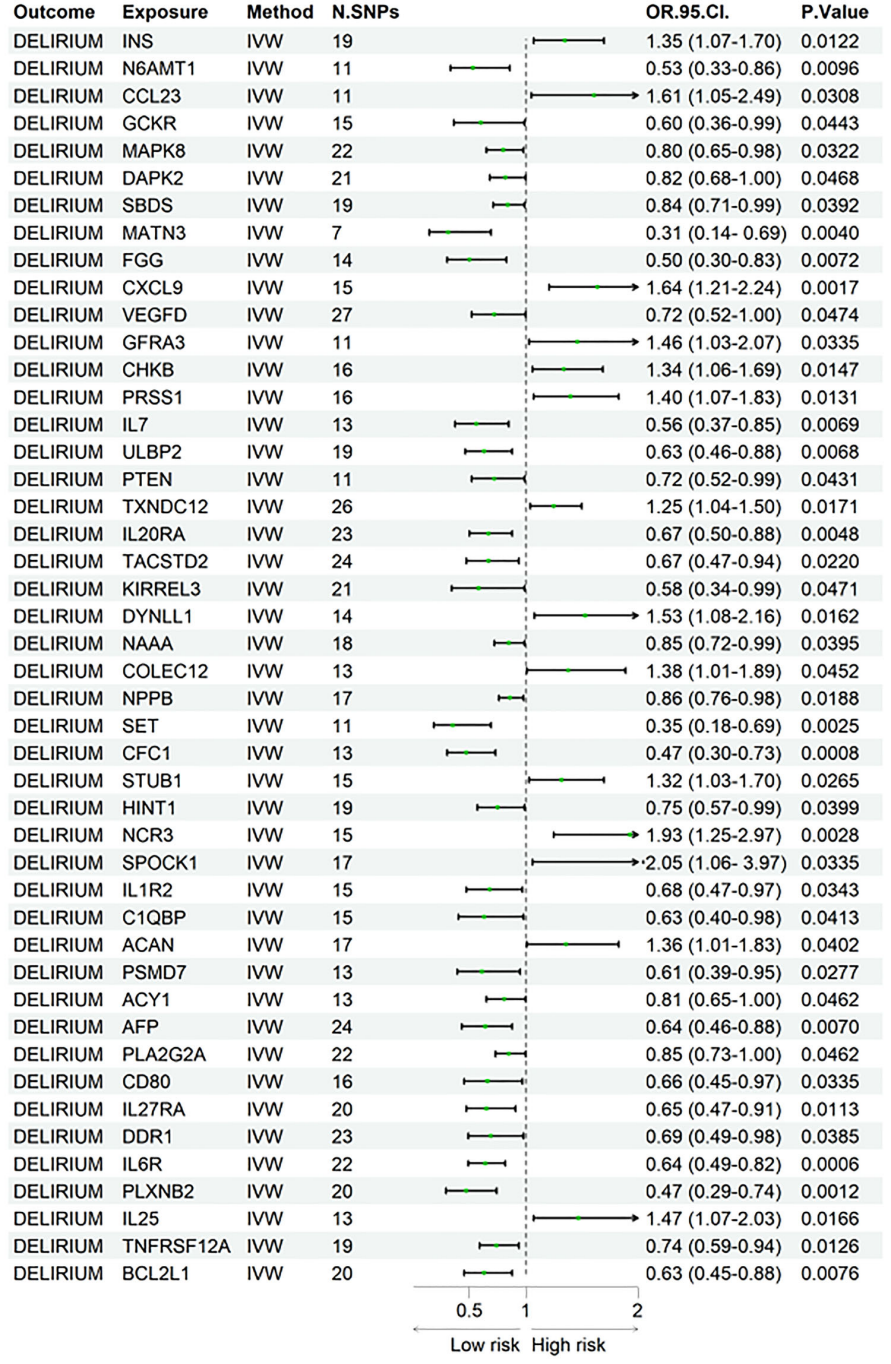
Forest plot of CSF proteins and delirium associations.

We employed the MR method to examine the genetic associations between CSF proteins and delirium. The analysis identified 46 CSF proteins genetically linked to delirium (*p* < 0.05). Among these, 14 proteins were positively associated with an increased risk of delirium, while the remaining 32 were negatively associated with delirium risk. The heterogeneity test showed no significant Q value (*p* > 0.05), indicating the absence of heterogeneity in the causal relationships between these proteins and delirium. Additionally, leave‐one‐out analysis affirmed the robustness of the results. Specifically, genetic variation in INS was significantly associated with an increased risk of delirium (OR = 1.35, 95% CI: 1.07–1.70, *p* = 0.01), while genetic variation in IL‐7 and BCL2L1 was significantly associated with a reduced risk of delirium (IL‐7: OR = 0.56, 95% CI: 0.37–0.85, *p* = 0.01; BCL2L1: OR = 0.63, 95% CI: 0.45–0.88, *p* = 0.01). Table 1 provides a summary of the sensitivity, heterogeneity, and pleiotropy tests performed in the MR analysis. Figure 2 displays a forest plot illustrating the strength and statistical significance of the associations between the various CSF proteins and delirium.

**TABLE 1:** Sensitivity analysis, heterogeneity, and pleiotropy tests in MR analysis.

Outcome	Exposure	Method	*β*	*p* value	OR(95%CI)	*Q*_pval	egger_intercept
Delirium	INS	IVW	0.30	0.01	1.35 (1.07–1.70)	0.07	0.08
Delirium	N6AMT1	IVW	−0.63	0.01	0.53 (0.33–0.86)	0.14	0.68
Delirium	CCL23	IVW	0.48	0.03	1.61 (1.05–2.49)	0.15	0.08
Delirium	GCKR	IVW	−0.52	0.04	0.60 (0.36–0.99)	0.21	0.19
Delirium	MAPK8	IVW	−0.22	0.03	0.80 (0.65–0.98)	0.19	0.82
Delirium	SBDS	IVW	−0.18	0.04	0.84 (0.71–0.99)	0.24	0.67
Delirium	DAPK2	IVW	−0.19	0.05	0.82 (0.68–1.00)	0.23	0.15
Delirium	MATN3	IVW	−1.17	0.00	0.31 (0.14–0.69)	0.26	0.67
Delirium	FGG	IVW	−0.70	0.01	0.50 (0.30–0.83)	0.29	0.20
Delirium	CXCL9	IVW	0.50	0.00	1.64 (1.21–2.24)	0.29	0.88
Delirium	VEGFD	IVW	−0.32	0.05	0.72 (0.52–1.00)	0.34	0.45
Delirium	GFRA3	IVW	0.38	0.03	1.46 (1.03–2.07)	0.32	0.28
Delirium	CHKB	IVW	0.29	0.01	1.34 (1.06–1.69)	0.45	0.06
Delirium	PRSS1	IVW	0.34	0.01	1.40 (1.07–1.83)	0.46	0.46
Delirium	IL7	IVW	−0.58	0.01	0.56 (0.37–0.85)	0.45	0.27
Delirium	ULBP2	IVW	−0.45	0.01	0.63 (0.46–0.88)	0.47	0.83
Delirium	PTEN	IVW	−0.33	0.04	0.72 (0.52–0.99)	0.49	0.69
Delirium	IL20RA	IVW	−0.41	0.00	0.67 (0.50–0.88)	0.49	0.73
Delirium	TXNDC12	IVW	0.22	0.02	1.25 (1.04–1.50)	0.50	0.15
Delirium	TACSTD2	IVW	−0.41	0.02	0.67 (0.47–0.94)	0.51	0.69
Delirium	KIRREL3	IVW	−0.54	0.05	0.58 (0.34–0.99)	0.50	0.86
Delirium	DYNLL1	IVW	0.42	0.02	1.53 (1.08–2.16)	0.48	0.65
Delirium	NAAA	IVW	−0.17	0.04	0.85 (0.72–0.99)	0.48	0.33
Delirium	COLEC12	IVW	0.32	0.05	1.38 (1.01–1.89)	0.54	0.74
Delirium	NPPB	IVW	−0.15	0.02	0.86 (0.76–0.98)	0.54	0.43
Delirium	SET	IVW	−1.05	0.00	0.35 (0.18–0.69)	0.52	0.76
Delirium	CFC1	IVW	−0.76	0.00	0.47 (0.30–0.73)	0.52	0.79
Delirium	STUB1	IVW	0.28	0.03	1.32 (1.03–1.70)	0.63	0.30
Delirium	HINT1	IVW	−0.29	0.04	0.75 (0.57–0.99)	0.65	0.74
Delirium	NCR3	IVW	0.66	0.00	1.93 (1.25–2.97)	0.65	0.79
Delirium	SPOCK1	IVW	0.72	0.03	2.05 (1.06–3.97)	0.68	0.19
Delirium	IL1R2	IVW	−0.39	0.03	0.68 (0.47–0.97)	0.69	0.95
Delirium	C1QBP	IVW	−0.47	0.04	0.63 (0.40–0.98)	0.69	0.25
Delirium	ACAN	IVW	0.31	0.04	1.36 (1.01–1.83)	0.73	0.71
Delirium	PSMD7	IVW	−0.50	0.03	0.61 (0.39–0.95)	0.72	0.08
Delirium	ACY1	IVW	−0.21	0.05	0.81 (0.65–1.00)	0.71	0.47
Delirium	AFP	IVW	−0.45	0.01	0.64 (0.46–0.88)	0.75	0.68
Delirium	PLA2G2A	IVW	−0.16	0.05	0.85 (0.73–1.00)	0.75	0.93
Delirium	CD80	IVW	−0.42	0.03	0.66 (0.45–0.97)	0.81	0.89
Delirium	IL27RA	IVW	−0.42	0.01	0.65 (0.47–0.91)	0.82	0.72
Delirium	DDR1	IVW	−0.37	0.04	0.69 (0.49–0.98)	0.83	0.20
Delirium	IL6R	IVW	−0.45	0.00	0.64 (0.49–0.82)	0.84	0.51
Delirium	PLXNB2	IVW	−0.76	0.00	0.47 (0.29–0.74)	0.90	0.74
Delirium	IL25	IVW	0.39	0.02	1.47 (1.07–2.03)	0.89	0.74
Delirium	TNFRSF12A	IVW	−0.30	0.01	0.74 (0.59–0.94)	0.92	0.79
Delirium	BCL2L1	IVW	−0.46	0.01	0.63 (0.45–0.88)	0.96	0.32

Table 1 presents sensitivity analysis, heterogeneity, and pleiotropy test results from a MR study on delirium. It identifies 46 CSF plasma proteins genetically associated with delirium, each showing *p*‐value < 0.05.

Figure 2 displays a forest plot showing the associations between CSF plasma proteins and delirium risk in MR. The plot includes ORs, 95% CIs, and *p*‐values. Most proteins have OR > 1 and *p *< 0.05, suggesting significant genetic links to delirium susceptibility.

**FIGURE 3:**
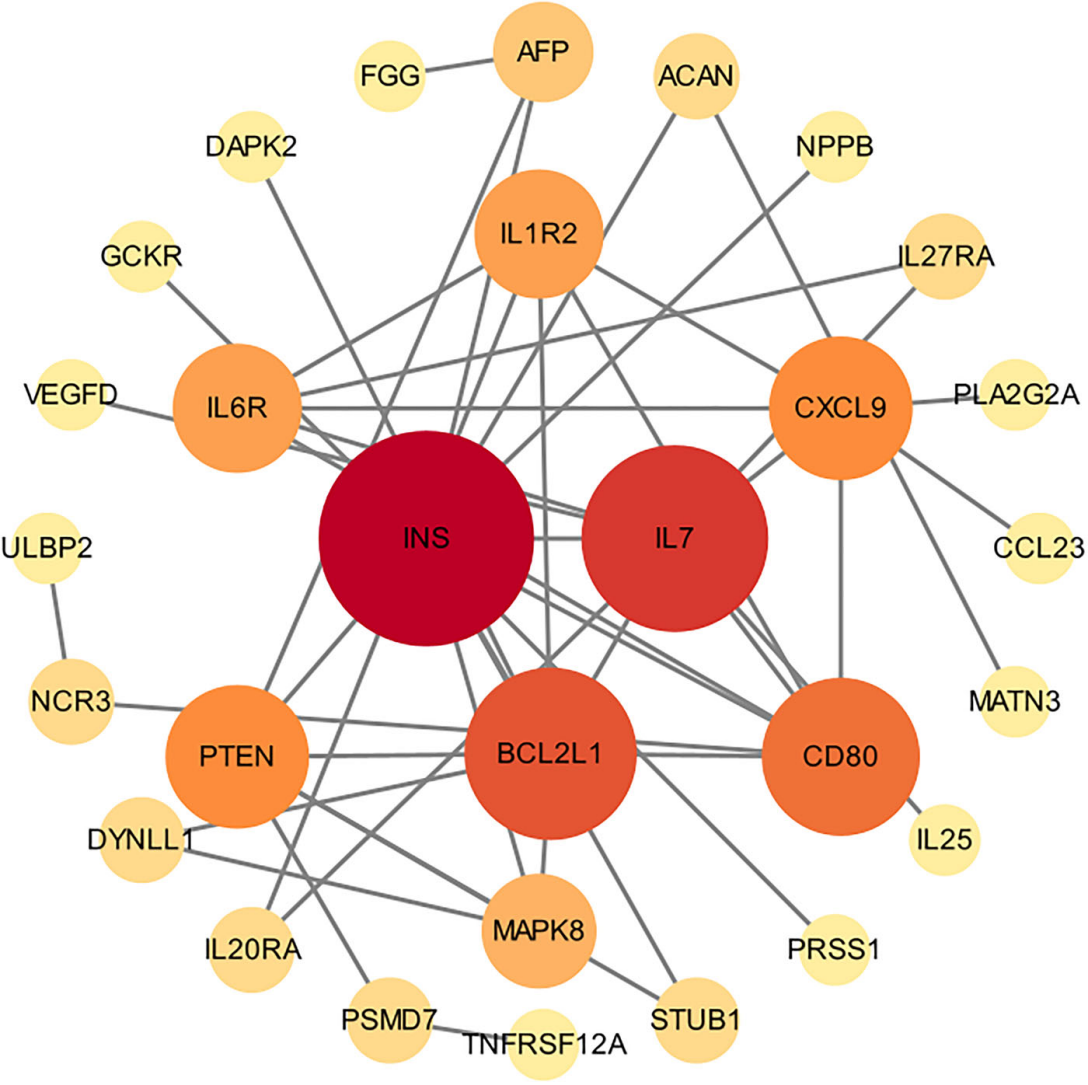
The PPI network.

Cytoscape 3.2. software was used to construct a PPI network consisting of three nodes and four edges (Figure 3), with the aim of revealing key molecular interactions among CSF proteins in delirium. Network analysis revealed several central nodes, including INS, BCL2L1, and IL‐7, which exhibited high connectivity, suggesting that these CSF proteins may play a central role in the pathophysiology of delirium. Notably, insulin (INS) has emerged as a key interacting protein. Figure 3 presents the PPI network, confirming INS, IL‐7, and BCL2L1 as critical proteins involved in the pathological process of delirium.

This protein‐protein interaction network map highlights key proteins associated with delirium. Larger nodes like INS, IL7, and BCL2L1 suggest central roles in the delirium pathway. Connections between nodes indicate known interactions, with red possibly denoting stronger associations with delirium. This visualization aids in pinpointing critical biomarkers and therapeutic targets in delirium.

### 3.1 Mendelian Randomization Analysis Results for Three Key Proteins

**FIGURE 4:**
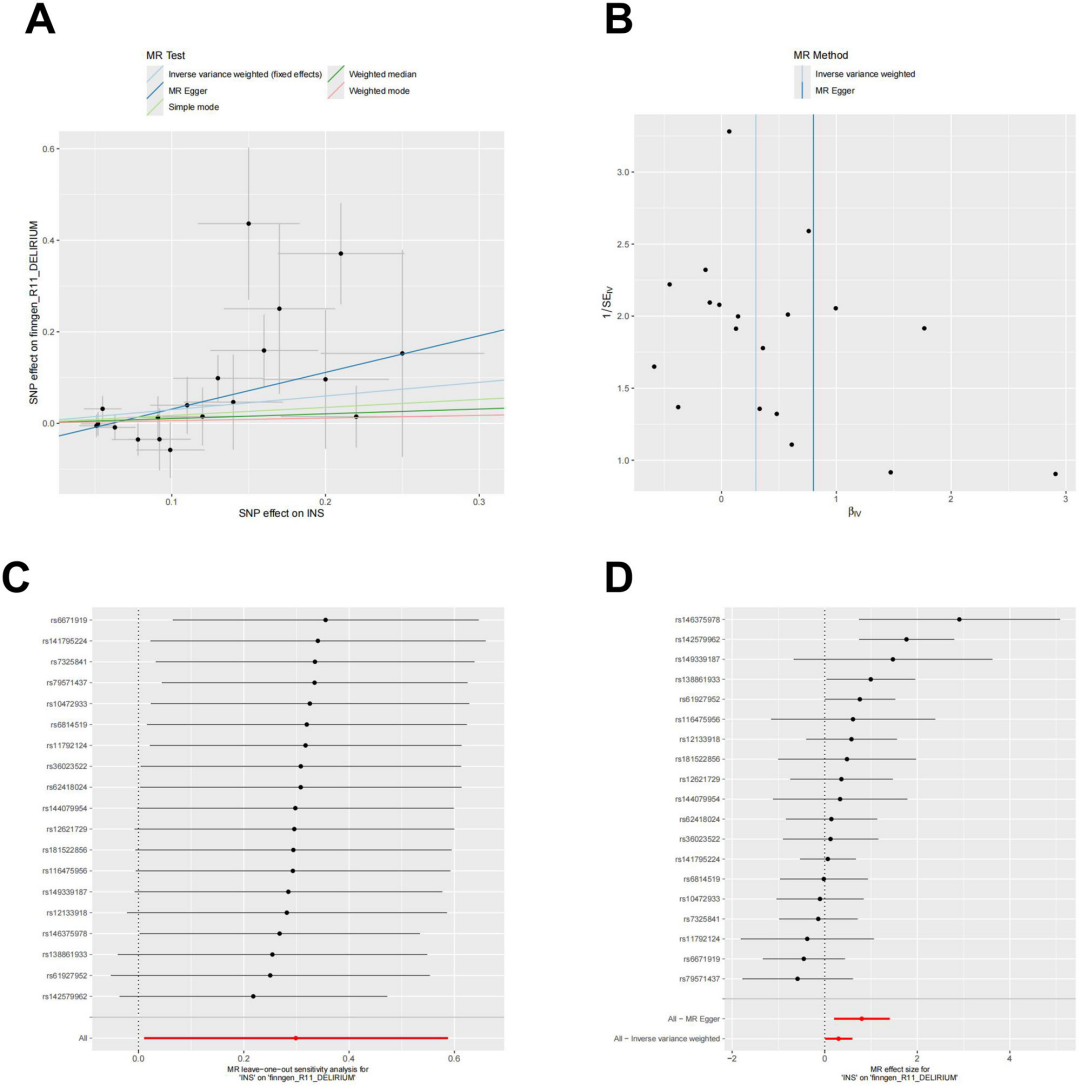
Mendelian randomization analysis of cerebrospinal fluid protein INS.

The MR analysis results for the CSF protein INS are summarized in Figure 4. Figure 4A: Scatter plot: The scatter plot demonstrates a positive linear trend for INS, indicating that elevated insulin expression in cerebrospinal fluid is associated with an increased likelihood of delirium. The slope of the line represents the causal effect for each method. The results revealed a positive correlation between CSF INS and delirium (*p =* 0.01, odds ratio [OR] [95% confidence interval (CI)] = 1.35 [1.07–1.70]). Figure 4B: Funnel plot: The funnel plot reveals a symmetric distribution of SNPs, suggesting no heterogeneity in the association. It assesses the randomness of the instrumental variables (IVs) and visually confirms the balanced distribution of IVs on either side of the IVW line, supporting the validity of the MR analysis in accordance with MR grouping principles. Figure 4C: Leave‐one‐out sensitivity analysis plot: The sensitivity analysis revealed that the association was not influenced by any individual SNP, indicating that the genetic association between CSF INS and delirium was stable, with no significant outliers affecting the data. Figure 4D: Forest plot: The forest plot was used to evaluate the predictive power of each SNP locus relative to the exposure and outcome. The solid points are predominantly located on the right, indicating that, according to the IVW method, an increase in CSF (INS) is associated with a greater risk of delirium. Additionally, the Cochrane's Q test results revealed no significant evidence of heterogeneity (*p =* 0.07> 0.05).

**FIGURE 5:**
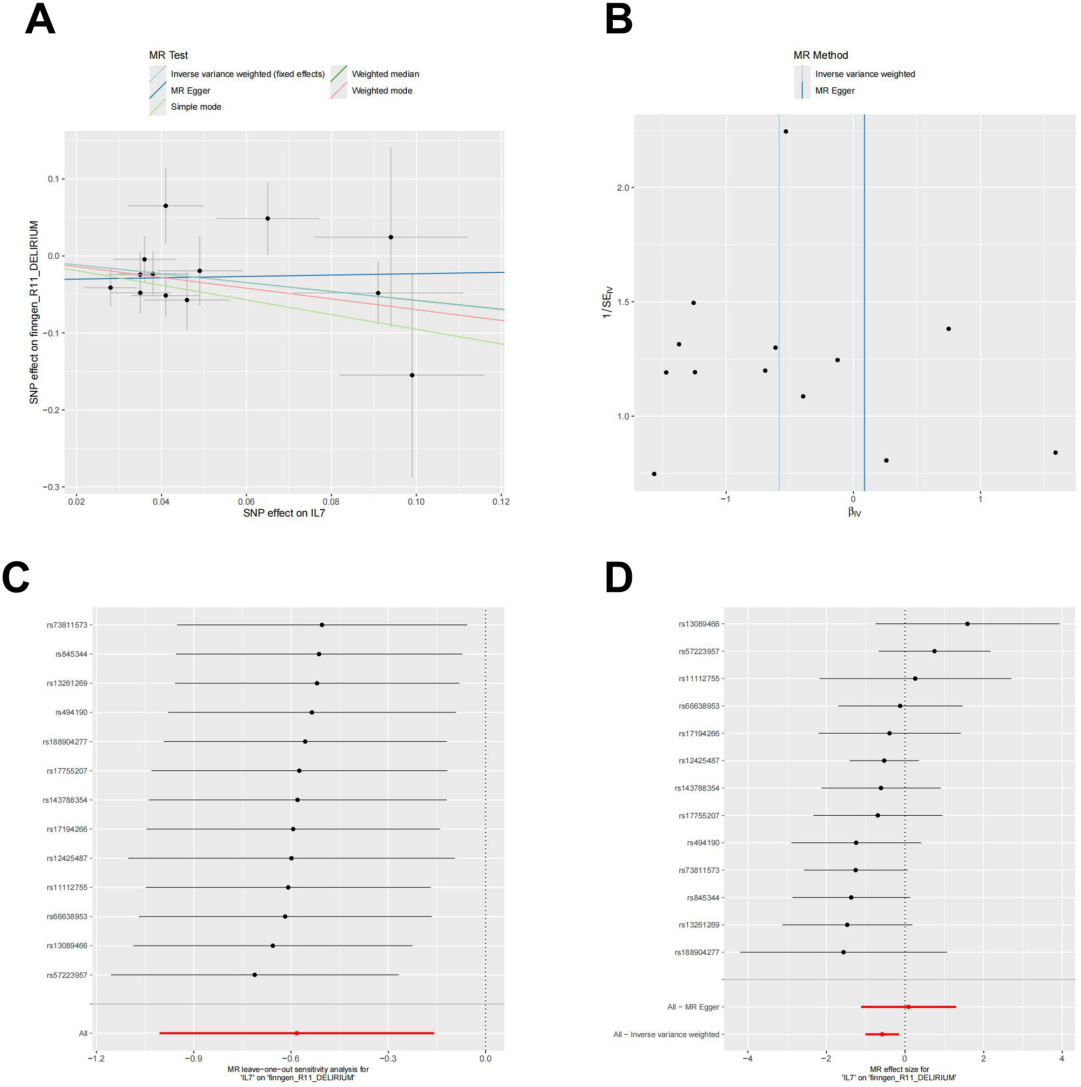
Mendelian randomization analysis of cerebrospinal fluid IL‐7.

**FIGURE 6:**
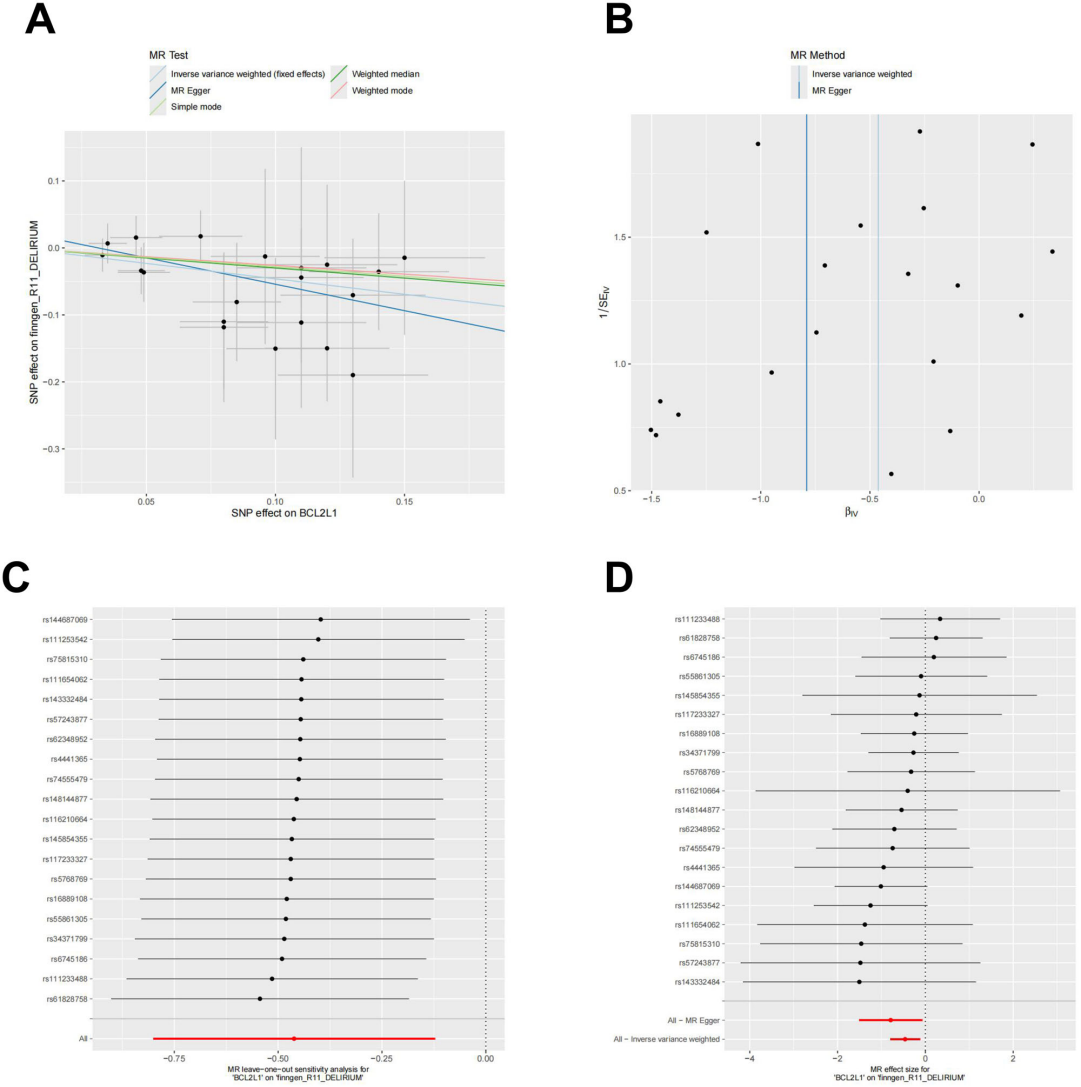
Mendelian randomization analysis of cerebrospinal fluid BCL2L1.

The MR analysis results for the CSF protein IL‐7 and BCL2L1 are summarized in Figures 5 and 6: In Figures 5A and 6A, the scatter plots support the finding that IL‐7 and BCL2L1 are negatively correlated with delirium. Figures 5B and 6B show symmetric SNP distributions (IVW), suggesting no heterogeneity in the associations. The forest plots are shown in Figures 5D and 6D show that most individual SNP effects cross zero, indicating weak single SNP effects, the overall effect remains significant. Subsequent leave‐one‐out tests confirmed the stability of these results.

Figure 4 presents an MR analysis of CSF insulin and delirium. It includes (A) scatter, (B) funnel, (C) forest, and (D) MR‐Egger plots to assess genetic effects, publication bias, effect estimates, and pleiotropy bias, evaluating the causal relationship between insulin and delirium risk.

Figure 5 utilizes these charts in an MR study to illustrate the role of CSF IL‐7 in the occurrence of delirium and to verify the robustness of this relationship.

Figure 6 utilizes these charts in an MR study to illustrate the role of CSF BCL2L1 in the occurrence of delirium and to verify the robustness of this relationship.

### 3.2 Enrichment Analysis Results

**FIGURE 7:**
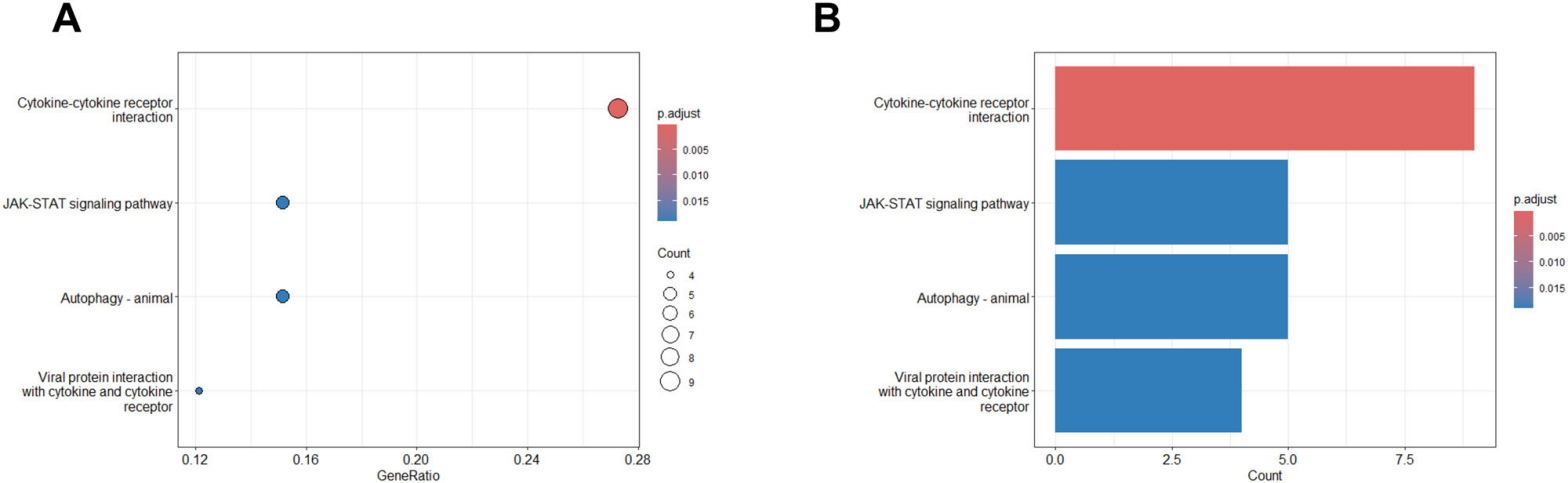
KEGG enrichment analysis of identified proteins in delirium pathways.

KEGG enrichment analysis was conducted on the 46 identified proteins via the protein knowledge base component of the UniProt database (https://www.uniprot.org/uniprot/) to explore their molecular and biological functions. Several pathways were found to be significantly enriched (*p* < 0.05), with the “cytokine‒cytokine receptor interaction” pathway showing the most significant enrichment. These pathways are involved in critical biological processes, such as the immune response, inflammatory response, and autophagy, providing valuable insights for further mechanistic investigations into delirium. The results are illustrated in Figure 7 (A: bubble chart, B: bar chart).

Figure 7 shows KEGG pathway enrichment. (A) Scatter plot highlights “Cytokine‐cytokine receptor interaction” as the most significant pathway. (B) Bar plot confirms it has the highest gene count and significance, with other notable pathways including JAK‐STAT signaling, autophagy, and viral protein interactions.

## 4 Discussion

This study is the first to utilize MR analysis to explore the causal relationship between CSF proteins and delirium. By integrating summary statistics from the ONTIME and FinnGen biobanks, we identified 46 protein‐coding genes with a causal link to delirium. Among the identified proteins, 14 CSF proteins were positively associated with delirium risk, whereas the remaining 32 were negatively associated. In the PPI network analysis, INS, BCL2L1, and IL‐7 were identified as central nodes, indicating their potential significant role in the pathophysiology of delirium. However, this finding does not necessarily suggest a stronger causal relationship between these proteins and delirium. The MR analysis results presented in Figure 2 show that the effect sizes of these proteins are not the most prominent. Therefore, further investigation is required to elucidate the precise causal connections between these proteins and delirium. Furthermore, subsequent KEGG enrichment analysis revealed that immune processes are likely involved in the potential pathophysiological mechanisms of delirium.

In recent years, research on the mechanisms underlying delirium has increasingly focused on neuroinflammation, synaptic damage, and blood‐brain barrier disruption (Alam et al. 2018). In accordance with the “neuroinflammation hypothesis” (Cerejeira et al. 2010), Poljak et al. (2014) conducted the first proteomic analysis of CSF in patients with delirium in 2014, identifying multiple protein families significantly associated with this condition. These findings provide a novel perspective for understanding the pathological mechanisms of delirium. Dillon et al. reported that alterations in insulin (INS) levels in preoperative CSF were associated with delirium, suggesting that this may be linked to abnormal glucose metabolism (2023). Under normal conditions, central insulin receptors are primarily distributed in brain regions such as the hypothalamus and hippocampus (Sagües‐Sese et al. 2022). When insulin binds to its receptor, it regulates neuronal growth, neurotransmitter release, synaptic function, and the proliferation and differentiation of central cells through the activation of the PI3K/Akt and RAS‐MAPK signaling pathways (Alvarez et al. 2018; Yonamine et al. 2023). In the presence of surgical trauma or inflammatory conditions (Neumann et al. 2008), elevated peripheral insulin levels may increase insulin permeability into the CSF, leading to central insulin resistance. When insulin signaling is impaired in the brain (Akhtar and Sah 2020), it can result in various forms of neuronal damage. Specifically, inactivation of the PI3K/Akt pathway diminishes the antiapoptotic capacity of neurons, increasing their vulnerability to stress‐induced damage and neuronal dysfunction. Moreover, insulin resistance can decrease brain glucose metabolism, trigger inflammatory responses, and cause insufficient neuronal energy supply and neuroinflammation, thereby increasing the risk of cognitive decline and neuropsychiatric conditions, including delirium (Méndez‐Flores et al. 2024; Zhang et al. 2021). Consequently, restoring or enhancing central insulin signaling has been proposed as a potentially effective neuroprotective strategy and has become a prominent area of contemporary neuroscience research (Chen et al. 2022; Sedzikowska and Szablewski 2021).

BCL2L1 (B‐cell lymphoma 2‐like 1) is a critical antiapoptotic protein that prevents the release of cytochrome C from the mitochondrial membrane by interacting with proapoptotic proteins, thereby blocking apoptotic pathways and providing protection under stress conditions. Additionally, BCL2L1 regulates the G1‐to‐S phase transition in the cell cycle by modulating intracellular reactive oxygen species (ROS) levels, effectively delaying cell cycle progression. The BCL‐XL protein encoded by BCL2L1 plays a vital role in neuronal survival and the maintenance of neuronal functions (Li et al. 2020; Chong et al. 2020). Studies have shown that BCL‐XL inhibits apoptosis by binding to proapoptotic proteins such as Bax and Bak, preventing increased permeability of the mitochondrial outer membrane. Furthermore, BCL‐XL preserves the mitochondrial membrane potential, decreases excessive ROS production, and regulates the intracellular calcium ion concentration to prevent damage caused by calcium overload. These protective functions extend beyond the central nervous system to include the functional regulation of peripheral immune cells.

The neuroprotective effects of BCL‐XL are especially prominent in key brain regions, particularly the hippocampus and striatum. By regulating neuronal apoptosis and exerting protective functions, BCL‐XL maintains the stability of these regions (Jurkowski et al. 2020; Park et al. 2022). Moreover, in the substantia nigra, BCL‐XL plays a pivotal role in protecting dopaminergic neurons and reducing neuronal death in patients with Parkinson's disease (Park et al. 2019).

In immune cells (Li et al. 2020; Chong et al. 2020; Sun et al. 2022). BCL‐XL modulates survival and function, thereby influencing systemic inflammatory responses. Within the context of neuroinflammation, BCL‐XL mitigates excessive activation of microglial and astrocytic cells, reduces the release of proinflammatory factors, and alleviates neuroinflammation.

Given its neuroprotective role, BCL‐XL has been implicated in various central nervous system diseases, including Parkinson's disease and Alzheimer's disease (Raj et al. 2021; D'Orsi et al. 2014).

Our findings suggest that BCL2L1 expression in CSF may be negatively correlated with the occurrence of delirium. This correlation may be linked to its role in regulating inflammation and immune responses. However, research on the involvement of BCL2L1 in delirium remains limited, highlighting the need for further investigation.

IL‐7 (interleukin‐7) is a cytokine produced by various cell types, including stromal cells, dendritic cells, and epithelial cells. Structurally, it consists of four antiparallel α‐helices and binds to type I cytokine receptors. IL‐7 plays a vital role in the development and functional regulation of the immune system and is essential for the development, differentiation, and survival of lymphocytes. It holds significant potential in the diagnosis and treatment of tumors and immune‐related diseases (Fu et al. 2024; Winer et al. 2022; Marković and Savvides 2024). Recently, interest in the role of IL‐7 in CSF in central neurodegenerative diseases has increased (Gertje et al. 2023; Stampanoni Bassi et al. 2024; Bruno et al. 2024; Lokau and Garbers 2020). However, the role of IL‐7 in these diseases remains controversial. On the one hand, IL‐7 supports the survival and proliferation of T cells and other immune cells, aiding in immune function and tissue repair. On the other hand, IL‐7 may activate and sustain inflammatory responses, potentially exacerbating disease progression. Additionally, while IL‐7 may reduce neuronal death by inhibiting proapoptotic proteins, its inflammation‐mediated effects could further disrupt the neuronal microenvironment. This dual and contradictory mechanism renders the role of IL‐7 in central neurodegenerative diseases uncertain. Our preliminary findings suggest a potential negative correlation between IL‐7 levels and delirium, but further research is needed to validate these results.

In our study, KEGG enrichment analysis of the relevant proteins revealed the cytokine‐cytokine receptor interaction pathway as the most significantly enriched pathway. This pathway, a critical immune pathway, facilitates intercellular signal transduction via cytokines and their receptors. It modulates a wide range of physiological and pathological processes (Lokau and Garbers 2020; Ferro et al. 2021), including immune cell proliferation, differentiation, migration, and survival, as well as the regulation of microglial activity, synaptic plasticity, and neuroinflammation, all of which are key contributors to the pathogenesis of delirium. Among the 46 CSF proteins associated with delirium identified in our analysis, several are inflammation related, such as interleukin‐6 receptor (IL‐6R), interleukin‐1 receptor type 2 (IL‐1R2), tumor necrosis factor receptor superfamily member 12A (TNFRSF12A), and interleukin‐27 receptor alpha chain (IL‐27RA), all of which are cytokine receptors. These receptors bind to cytokines such as IL‐6 and IL‐1, activating proinflammatory signaling pathways, leading to microglial activation and exacerbated neuroinflammation. This heightened neuroinflammation disrupts the brain's microenvironment, impairing synaptic transmission and contributing to delirium. Furthermore, proteins such as vascular endothelial growth factor D (VEGFD) and C‐X‐C motif chemokine ligand 9 (CXCL9) influence blood‒brain barrier function through this pathway (Tan et al. 2019; Niu et al. 2019). VEGFD plays a pivotal role in angiogenesis and vascular permeability, whereas CXCL9, a chemokine, directs immune cell migration to inflammatory sites. These processes disrupt the blood‒brain barrier, intensify central nervous system inflammation, and further precipitate the onset of delirium.

The strengths of this study include the first report of the genetic relationship between CSF proteins and their encoding genes with delirium risk and the exploration and integration of the pathological mechanisms of delirium. However, our study has several limitations. First, in the GWAS analysis of delirium data, we focused on only one phenotype (Delirium, not induced by alcohol and other psychoactive substances.). Second, it should be noted that the participants in the MR analysis were exclusively from Europe, which may limit the generalizability of the results to individuals from other continents or different ethnic backgrounds. Future studies could expand the research findings to include other ethnic groups. Third, different tissues may exhibit distinct genetic regulatory mechanisms, and focusing solely on CSF proteins may not offer a comprehensive understanding of delirium. Future research should investigate the proteins and their encoding genes in blood and brain tissues to enhance our understanding of delirium. Additionally, given the relatively small sample size in this study (*n* = 970), the associations identified at the nominal significance threshold (*p* < 0.05) should be regarded as preliminary exploratory findings. Since we did not adjust for multiple comparisons, the risk of false positives remains. Therefore, we plan to validate these findings in larger‐scale studies to confirm their robustness and generalizability.

## 5 Conclusion

The study indicates that the levels of INS, BCL2L1, and IL‐7 in CSF may be linked to the risk of delirium, with INS playing a pivotal role. As this is an exploratory analysis and no adjustments were made for multiple comparisons, the findings should be regarded as preliminary and require validation in larger, more diverse cohorts. Future research should incorporate multi‐omics approaches to investigate the functional roles of these proteins in animal models, with particular attention to the cytokine‐cytokine receptor interaction pathway, which may play a crucial role in delirium pathogenesis and warrants further exploration.

## Author Contributions

**Zhihui Xu**: methodology, software, writing–original draft. **Fei Ye**: data curation, investigation, writing–review and editing. **Zhantang Yuan**: conceptualization, investigation, validation. **Chiyi Liu**: validation, formal analysis, supervision. **Simin Zhu**: visualization, project administration, writing–original draft. **Binfei Li**: funding acquisition, writing–review and editing. **Qibiao Wu**: funding acquisition, writing–review and editing.

## Ethics Statement

This study accessed data from the FinnGen and ONTIME databases, which are publicly available and have been ethically approved. No additional ethical approval was required for this analysis as it utilized existing, anonymized datasets. The study adheres to the Declaration of Helsinki and relevant data protection regulations.

## Conflicts of Interest

The authors declare no conflicts of interest.

## Peer Review

The peer review history for this article is available at https://publons.com/publon/10.1002/brb3.70836

## Data Availability

Data for this study were obtained from the 11th iteration of the FinnGen Biobank, accessible through the Finnish Institute for Health and Welfare (THL) upon approval. For access, visit FinnGen. The ONTIME platform (https://ontime.wustl.edu) at Washington University in St. Louis is accessible after registration and agreement to data use terms. Researchers should follow the respective database protocols for data access.
